# Lessons Learned in a Community-Based, Randomized Controlled Trial of a Complicated Grief Intervention

**DOI:** 10.1093/geroni/igaa057.2453

**Published:** 2020-12-16

**Authors:** Harleah Buck, Paula Cairns, Nnadozie Emechebe, Diego Hernandez, Tina Mason, Jesse Bell, Kevin Kip, Cindy Tofthagen

**Affiliations:** 1 University of South Florida, Tampa, Florida, United States; 2 University of south florida, Tampa, Florida, United States; 3 Mayo Clinic, Jacksonville, Florida, United States

## Abstract

Complicated grief (CG), severe, prolonged (>12 months) grieving, disproportionately affects older adults. A prospective two-group, waitlisted RCT examined whether four sessions of Accelerated Resolution Therapy (ART) was effective in informal caregivers by comparing pre-to-post ART changes and investigating variation in treatment response by baseline CG levels. Inclusion: ≥60 years, Inventory of Complicated Grief >25. Paired t-tests of mean (SD) differences compared pre- to post-ART; pre-ART to 8-week follow-up, and post-ART to 8-week follow-up; then stratified by median baseline level of CG. Mean (SD) age of 54 participants was 68.7 (7.2) years, 85% female, and 93% white. Significantly greater CG reduction (-22.8 (10.3)) vs. waitlist (-4.3 (6.0)) was found. Within-participant effect sizes from baseline to 8-weeks post treatment were 1.96 (95% CI: 1.45, 2.47; p<0.0001). Treatment effects did not substantially differ by baseline levels. Lesson learned was that it was possible to successfully recruit and treat CG in the community.

